# Changes in the Expression of Transcription Factors Involved in Modulating the Expression of EPO-R in Activated Human CD4-Positive Lymphocytes

**DOI:** 10.1371/journal.pone.0060326

**Published:** 2013-04-05

**Authors:** Katarzyna A. Lisowska, Joanna E. Frackowiak, Anna Mikosik, Jacek M. Witkowski

**Affiliations:** Department of Pathophysiology, Medical University of Gdańsk, Gdańsk, Poland; Virgen Macarena University Hospital, School of Medicine, Spain

## Abstract

We have recently described the presence of the erythropoietin receptor (EPO-R) on CD4^+^ lymphocytes and demonstrated that its expression increases during their activation, reaching a level reported to be typical for erythroid progenitors. This observation suggests that EPO-R expression is modulated during lymphocyte activation, which may be important for the cells’ function. Here we investigated whether the expression of GATA1, GATA3 and Sp1 transcription factors is correlated with the expression of EPO-R in human CD4^+^ lymphocytes stimulated with monoclonal anti-CD3 antibody. The expression of GATA1, GATA3 and Sp1 transcription factors in CD4^+^ cells was estimated before and after stimulation with anti-CD3 antibody by Western Blot and flow cytometry. The expression of EPO-R was measured using real-time PCR and flow cytometry. There was no change in the expression of GATA1 and GATA3 in CD4^+^ lymphocytes after stimulation with anti-CD3 antibody. However, stimulation resulted in the significantly increased expression of the Sp1 factor. CD4^+^ lymphocytes stimulated with anti-CD3 antibody exhibited an increase in both the expression level of *EPOR* gene and the number of EPO-R molecules on the cells’ surface, the latter being significantly correlated with the increased expression of Sp1. Sp1 is noted to be the single transcription factor among the ones studied whose level changes as a result of CD4^+^ lymphocyte stimulation. It seems that Sp1 may significantly affect the number of EPO-R molecules present on the surface of activated CD4^+^ lymphocytes.

## Introduction

The erythropoietin receptor (EPO-R) first appears on the surface of cells in the early stages of erythropoiesis known as the erythroid colony-forming unit (CFU-E) and erythroid burst-forming unit (BFU-E), thus enabling erythropoietin (EPO) to regulate the cells’ proliferation and differentiation in response to low oxygen concentration in the body [1]. Expression of EPO-R has also been reported in many non-hematopoietic cells including cardiomyocytes, neuronal cells, endothelial cells, T and B lymphocytes and monocytes, but its effect on these cells is not as clear as its role in erythropoiesis. What’s more, the number of EPO-R molecules in the above-mentioned cells can vary, e.g. 27000 molecules per cell in endothelial cells [2], or as low as 100 molecules per cell in T lymphocytes [3]. However, stimulation of CD4^+^ lymphocytes with anti-CD3 antibody results in the increased number of EPO-R molecules which may reach 1000 molecules per cell [3], [4], a value reported to be typical for erythroid progenitors [5]. This observation suggests that EPO-R expression is modulated during lymphocyte activation, thus emphasizing its significance in the function of these cells. In fact, we have shown that in many different aspects EPO influences these cells in hemodialyzed (HD) patients treated with recombinant human erythropoietin (rhEPO). These include features such as cytokine production and phenotype of CD4^+^ lymphocytes [6], [7], [8], [9], [10]. Moreover, we have demonstrated that rhEPO can directly affect CD4^+^ lymphocytes by increasing CD95 expression [4], which could be one of the mechanisms by which rhEPO modulates T lymphocytes’ responses in HD patients.

The expression of the *EPOR* gene in erythroid cells is regulated by the GATA family of transcription factors (mainly GATA1) and by Sp1, which belongs to the Sp/KLF family of transcription factors [11]. GATA transcription factors are divided into two main groups depending on their tissue distribution: GATA1, GATA2 and GATA3 are expressed mainly in hematopoietic cells, while GATA4, GATA5 and GATA6 are found in endoderm-derived tissues and organs (reviewed in [12]). Expression of GATA1 is very low in progenitor cells but increases noticeably when cells are induced to differentiate into the erythroid lineage [13] and is responsible for transcriptional regulation of the majority of erythroid genes including the *EPOR* gene [14]. GATA1 expression has not yet been reported in either T lymphocytes or T-cell lines. Meanwhile, GATA3 expression is mainly associated with the development and differentiation of the T-cell lineage [15]. There are several GATA binding sites within the promoter of the *EPOR* gene; these can be recognized by not only GATA1 but also GATA2, and GATA3, as demonstrated in various neuronal cells [16]. This is due to the fact that GATA family factors bind to the DNA consensus sequence T/A (GATA) A/G and its alternatives [17].

The level of *EPOR* gene expression in the erythroid cells does not depend on GATA alone but is also regulated by Sp1 [11]. Binding of Sp1 to a mutated promoter sequence of the *EPOR* gene results in a marked decrease in the gene’s transcriptional activity, underlining the significance of this transcription factor in regulating the expression of EPO-R [11]. Moreover, it seems that *EPOR* gene expression is regulated differently by GATA and Sp1 transcription factors in various cell types. For example, overexpression of GATA factors in neuronal cells has no significant effect on the expression of *EPOR* mRNA [16]. In myoblasts the *EPOR* gene is co-expressed with the *GATA3* gene [18]. Mutations within the promoter region of the *EPOR* gene in the site recognized by Sp1 result in a significant reduction of the gene’s activity, as shown in the erythroleukemia cell line K562 [11].

However, the expression and function of GATA and Sp1 factors in helper T lymphocytes (Th cells, CD4^+^ lymphocytes) is still poorly understood. GATA3 is considered to be the only GATA member that is expressed in CD4^+^ lymphocytes (reviewed in [12]). The transcriptional activity of Sp1 has been described in proliferating T lymphocytes and is presumably involved in cell cycle regulation [19]. Therefore, we examined whether the expression of GATA1, GATA3 and Sp1 transcription factors, which are considered to be paramount in the regulation of EPO-R expression, is correlated with the expression of EPO-R in human CD4^+^ cells stimulated with immobilized monoclonal anti-CD3 antibody. The advantage of such an experiment is the fact that the conditions used are similar to physiological conditions as they mimic the binding of antigen presenting cells (APCs) to lymphocytes. Western Blot and flow cytometry techniques were used to investigate the expression of the above-mentioned transcription factors in activated CD4^+^ lymphocytes of healthy individuals. We also determined the number of EPO-R molecules per cell and the expression of the *EPOR* gene.

## Materials and Methods

The study was performed with the use of venous blood drawn from 11 healthy volunteers (mean age 33.00±10.61 years), 6 women and 5 men. 40 ml of venous peripheral blood from each individual was collected in tubes containing EDTA as the anti-coagulant agent. All participants were informed about the purpose of the tests and gave their written informed consent; the study has been approved by the Bioethical Committee for Scientific Research at the Medical University of Gdansk.

### Stimulation of Peripheral Blood Mononuclear Cells and Isolation of CD4^+^ Lymphocytes

Peripheral blood mononuclear cells (PBMCs) were isolated by centrifugation through a Histopaque™ gradient (Sigma Chemical Co., USA). Cells were then stimulated with immobilized anti-CD3 antibody (125 ng/ml) and incubated for three days at 37°C, 5% CO_2_. CD4^+^ cells were magnetically separated according to the manufacturer’s instructions using the CD4 Negative Isolation Kit (Dynal Biotech ASA, Norway) at four separate time intervals, i.e. before stimulation (*ex vivo*) as well as 24, 48, and 72 hours following stimulation with anti-CD3 antibody. The purity of CD4^+^ cells was established by flow cytometric analysis. Cells were then frozen in liquid nitrogen and stored at −80°C.

### Cytometric Analysis of EPO-R and Sp1 Expression in CD4^+^ Lymphocytes

Cytometric procedures needed to determine the surface expression of EPO-R and Sp1 *ex vivo* were performed as follows. Red blood cells (RBC) were removed from cell suspension using a lysis buffer containing 0.8% NH_4_Cl and 0.1% KHCO_3_ in distilled water. Cells were then washed with PBS (phosphate buffered saline) buffer and stained with antibodies as described below. Estimation of the intracellular expression of Sp1 in CD4^+^ cells was carried out by fixing cells with pre-warmed BD Cytofix™ Buffer (Becton Dickinson, USA) for 15 minutes at 37°C followed by a permeabilization procedure in which cells were incubated for 30 minutes at 4°C with BD Phosflow Perm Buffer III (Becton Dickinson, USA). After two wash steps with BD Pharmingen Stain Buffer (Becton Dickinson, USA) cells were either stained with FITC-conjugated anti-Sp1 antibody (Merck Millipore, Germany), the FITC-conjugated isotype control (eBioscience, USA) or RPE-Cy5-conjugated anti-CD4 antibody for 40 minutes at room temperature. After washing in BD Pharmingen Stain Buffer, cells were suspended in 200 µl of BD Pharmingen Stain Buffer followed by flow cytometric analysis. PBMCs stimulated with anti-CD3 antibody were collected following 24, 48, and 72 hours of cell culture, washed with PBS and stained according to the protocol described above. Cytometric analysis of the number of EPO-R molecules on the surface of CD4^+^ lymphocytes was performed as previously described [3].

### Western Blot Analysis of GATA1, GATA3 and Sp1 Expression in CD4^+^ Lymphocytes

Samples containing 200 000 magnetically separated CD4^+^ cells for each studied protein and for β-actin were boiled 8 minutes in the Laemmli buffer. Proteins in the samples were separated by polyacrylamide electrophoresis according to their molecular weight and then transferred from the gel to Immobilon-P transfer membrane (Millipore, USA) using the semi-dry transfer (Trans-Blot® SD, BioRad, USA). Membranes containing separated proteins were blocked with 3% non-fat milk in TRIS-buffered saline (TBS) and then incubated overnight under gentle agitation at +4°C separately with one of the monoclonal mouse primary antibodies against the following proteins (Abcam, USA): human Sp1 (1 mg/ml), GATA1, GATA3 (0.5 mg/ml) and β-actin (0.5 mg/ml). Appropriate peroxidase (HRP)-conjugated polyclonal rabbit anti-mouse Ig (Abcam, USA) at a concentration of 2 mg/ml and the ECL system (SuperSignal™ kit; Pierce, USA) were used to detect and visualize the proteins of interest. Detection and recording of specific bands was performed by exposing the membranes to photoradiographic film Medical X-Ray film (Primax RTG-B, Poland). The developed and fixed films were digitized using the GDS-8000 System and quantified using Labworks Image Acquisition software and Analysis Software Version 4.0 (UVP Bioimaging System, UK). Densitometric analysis was performed using the ImageJ 1.44 program (Wayne Rasband, National Institutes of Healthy, USA). Relative amounts of transcription factors were expressed as arbitrary densitometric units after standardization vs. β-actin content.

### Nested PCR Estimation of *EPOR* Gene Expression

Total RNA from samples containing 600 000 magnetically separated CD4^+^ cells each was isolated using TriReagent (Sigma Chemical Co., USA) according to the manufacturer’s protocol and immediately converted to cDNA using the Improm-II Reverse Transcription System (Promega, USA). Amplification of *EPOR* and *GAPDH* gene products was performed by nested polymerase chain reaction (nested PCR) using 1 µl of cDNA in the first PCR run and 2 µl of this obtained product in the second PCR run as template. The following primers (BLIRT, Poland) were used: 5′-GGTTGGAGGACTTGGTGTGT-3′, 5′-GAGACGTCATGGGTGTCTCA-3′ (outer primers, product size 363 bp), 5′-GGGCAACTACAGCTTCTCCT-3′, 5′-TCATTGATGTGGATGACACG-3′ (inner primers, product size 201 bp) for *EPOR*; 5′-TGCTGATGATCTTGAGGCTG-3′ and 5′-GGCGTCTTCACCACCATGG-3′ (product size 150 bp) for *GAPDH.* 30 cycles were included in the first and 20 in the second PCR reaction. Each cycle consisted of 30 seconds of denaturation at 94°C, 30 seconds of annealing at 59°C and 30 seconds of extension at 72°C. The PCR reactions were run on the Personal Cycler (Eppendorf, Germany). Amplified PCR products were run on 2% agarose gel and visualized by staining with ethidium bromide.

### Real-time PCR Quantification of *EPOR* Gene Expression

LightCycler and FastStart DNA Master SYBR Green I Kit (Roche Diagnostics, Germany) were used for real-time PCR quantification and the following primers (BLIRT, Poland) were chosen: 5′-CTAGAGTTGCGCGTCACAG-3′ and 5′-GGCGTCTAGGAGCACTACTT-3′ for *EPOR*, 5′-CAGTCAGCCGCATCTTCTTT-3′ and 5′-GACCAAATCCGTTGACTCCG-3′ for *GAPDH*. The reaction was performed starting with 10 minutes of activation at 95°C, followed by 40 cycles composed of 10 seconds of denaturation at 95°C, 10 seconds of annealing at 59°C and 4 seconds of extension at 72°C and finally 30 seconds of cooling at 40°C. Results obtained from *EPOR* gene product quantification were calculated on the basis of the standard curve using LightCycler Software 4.05 (Roche Diagnostics, Germany).

### Analysis and Statistics

**Figure 1:**
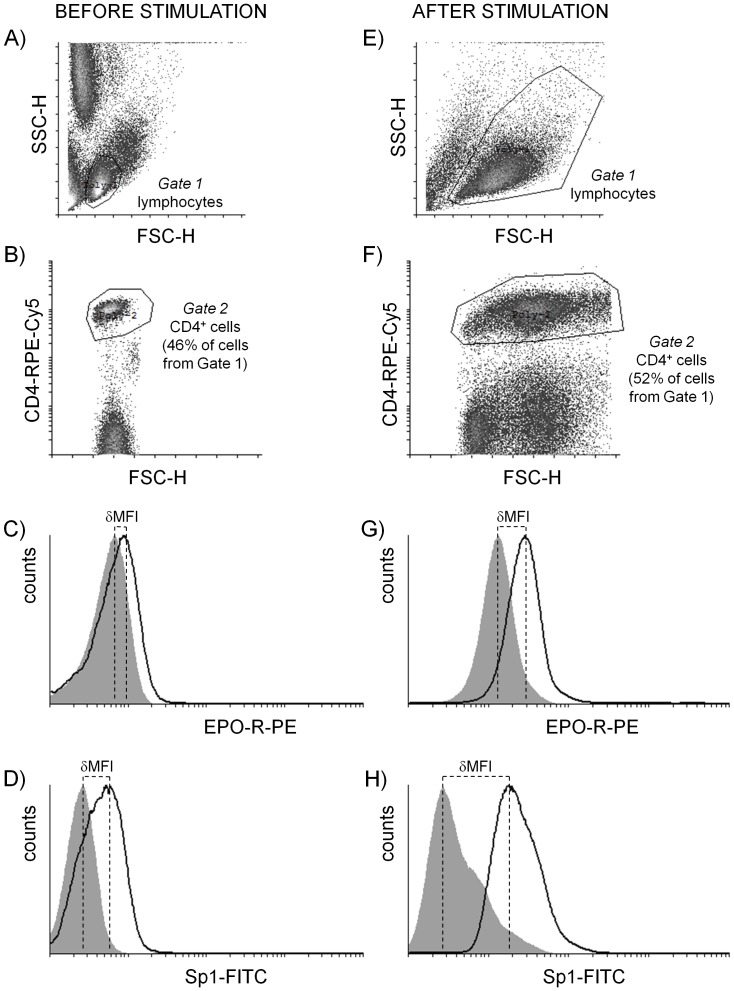
Fluorescence analysis of EPO-R and Sp1 expression measured by flow cytometry in CD4^+^ lymphocytes before and after stimulation with anti-CD3 antibody. CD4^+^ cells were selected on the basis of forward and side scatter characteristics of lymphocytes, *Gate 1* (A, E) and expression of CD4 antigen in gated lymphocytes, *Gate 2* (B, F). Figures C and D present the expression of EPO-R and Sp1 in CD4^+^ cells (cells from *Gate 1* and *Gate 2*) before stimulation, figures G and H present the expression of EPO-R and Sp1 in CD4^+^ cells stimulated with anti-CD3 antibody for 48 hours, respectively. For individual samples, expression of EPO-R and Sp1 was estimated as mean fluorescence shift (black line) toward isotype control (gray histogram): δMFI = MFI of the population of interest – MFI of the appropriate isotype control. Quantitative fluorescence analysis assessing the number of EPO-R molecules per cell based on δMFI was performed as previously described [3].

Fluorescence analysis was performed on FACScan (Becton Dickinson, USA). Data was analyzed using Cyflogic software version 1.2.1 (©Perttu Terho and ©CyFlow Ltd). The gating strategy was as follows: CD4^+^ cells were selected on the basis of the lymphocytes’ forward and side scatter characteristics (*Gate 1* in Fig. 1) and their expression of the CD4 antigen (*Gate* 2 in Fig.1). The expression of EPO-R and Sp1 in CD4^+^ cells (cells from *Gate 1* and *Gate 2*) is shown in a histogram (Fig. 1C, D, G, H). The expression of EPO-R and Sp1 in individual samples of CD4^+^ cells was measured as mean fluorescence intensity (MFI): δMFI = MFI of the population of interest – MFI of the appropriate isotype control. Quantitative fluorescence analysis assessing the number of EPO-R molecules per cell based on δMFI was performed as previously described [3].

Statistical analysis was carried out using Statistica software version 8.0 (StatSoft, Poland). Data was analyzed using non-parametric tests and the level of significance in all was p ≤ 0.05. Graphs were prepared using Statistica version 8.0 and GraphPad Prism version 6.01 (GraphPad Software, USA).

## Results

### Cytometric Evaluation of EPO-R and Sp1 Expression in Stimulated CD4^+^ Lymphocytes

**Figure 2:**
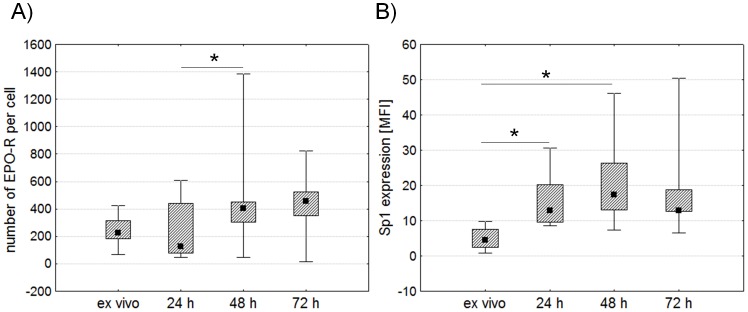
Comparison of EPO-R and Sp1 expression measured by flow cytometry in CD4^+^ lymphocytes before and after stimulation with anti-CD3 antibody. Figures A and B present changes in the number of EPO-R molecules per cell and the expression of Sp1, respectively. Midpoints of figures present medians, boxes present the 25 and 75 percentile and whiskers outside visualize the minimum and maximum of all the data, *p<0.05, Friedman ANOVA and Post Hoc test, MFI – mean fluorescence intensity.

**Figure 3:**
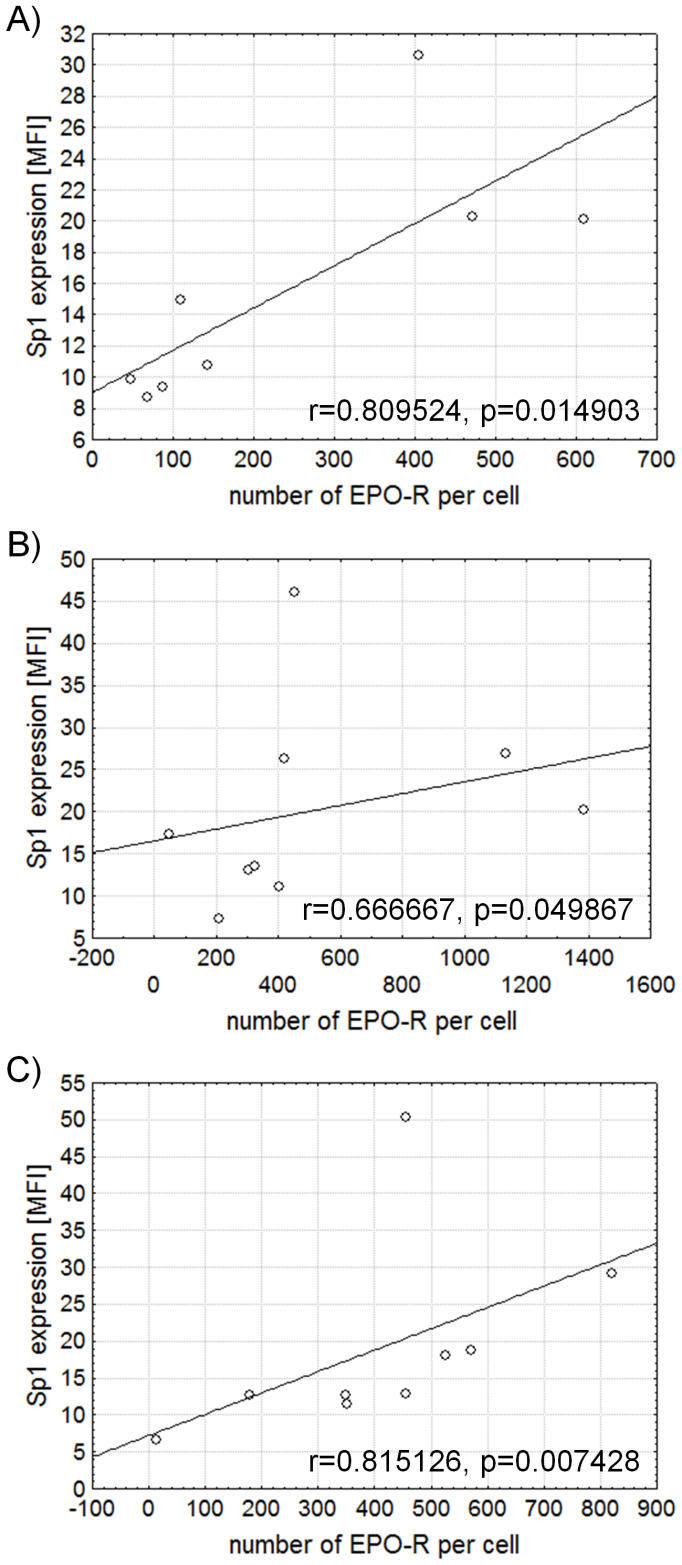
Correlations between expression of EPO-R and Sp1 measured by flow cytometry in CD4^+^ lymphocytes stimulated with anti-CD3 antibody. Graphs present correlations between the number of EPO-R molecules per cell and the expression of Sp1 following 24 (A), 48 (B) and 72 (C) hours of stimulation with anti-CD3 antibody. Figures present Spearman R correlation, MFI – mean fluorescence intensity.

We stimulated PBMCs with immobilized monoclonal anti-CD3 antibody to examine the expression of GATA1, GATA3, Sp1 and EPO-R in human CD4^+^ lymphocytes. The number of EPO-R molecules and the expression of Sp1 were determined using flow cytometry (Fig. 1). Through EPO-R expression analysis, we once again demonstrated that CD4^+^ lymphocytes present a low number of receptor molecules on their surface (mean value of 270 molecules per cell). However, as previously shown [3], [4], the number of molecules of EPO-R on the surface of CD4^+^ lymphocytes significantly increases 48 hours after stimulation with anti-CD3 antibody (Fig. 2A). At the same time we observed a significant increase in the expression of Sp1 factor in CD4^+^ lymphocytes (Fig. 2B). There was a positive correlation between the expression of Sp1 factor and the number of EPO-R molecules in stimulated CD4^+^ lymphocytes (Spearman correlation r = 0.809524, p = 0.014903 for 24 hours of stimulation; r = 0.666667, p = 0.049867 for 48 hours of stimulation and r = 0.815126, p = 0.007428 for 72 hours of stimulation) (Fig. 3). The strongest correlation between these parameters occurred on the 3rd day of stimulation with anti-CD3 antibody, when both EPO-R and Sp1 expression reach their highest level (Fig. 3C).

### Expression of GATA1, GATA3, and Sp1 in Stimulated CD4^+^ Lymphocytes by Western Blot

**Figure 4:**
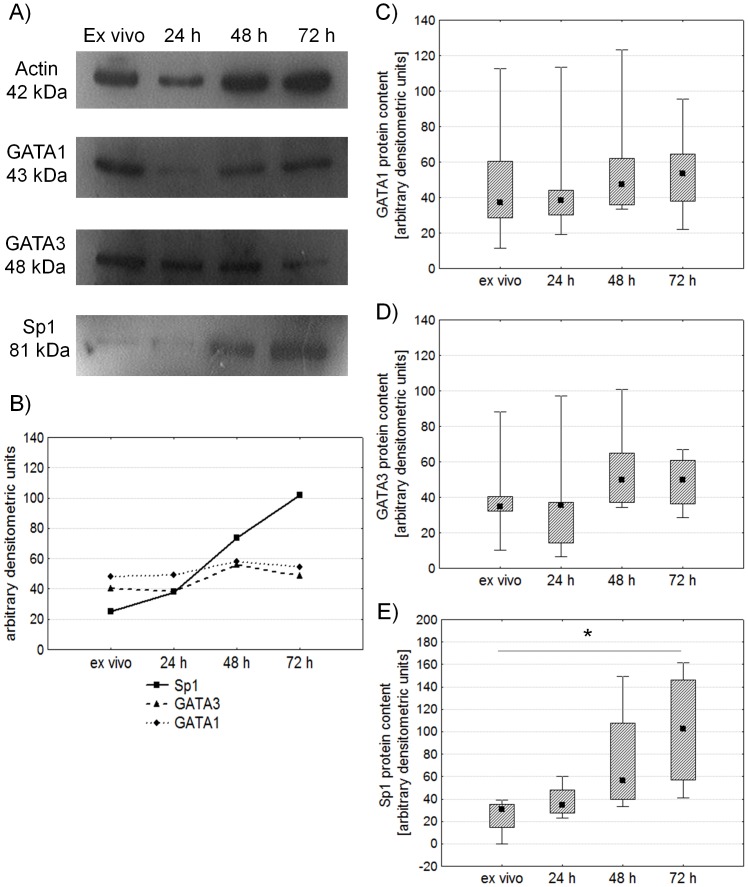
Comparison of GATA1, GATA3 and Sp1 expression determined by Western Blot in isolated CD4^+^ lymphocytes before and after stimulation with anti-CD3 antibody. Figure A shows representative results. Figure B presents mean expression of GATA1, GATA3 and Sp1 in arbitrary densitometric units. Figures C, D and E present expression of GATA1, GATA3 and Sp1, respectively. Midpoints of figures present medians, boxes present the 25 and 75 percentile and whiskers outside visualize the minimum and maximum of all the data, *p<0.05, Friedman ANOVA and Post Hoc test.

We also used the Western Blot method to detect transcription factors in magnetically separated CD4^+^ cells. We detected GATA1, GATA3 and Sp1 transcription factors in isolated CD4^+^ cells both *ex vivo* and after stimulation with anti-CD3 antibody (Fig. 4A). GATA1 and GATA3 expression measured as arbitrary densitometric units after standardization vs. β-actin content was at a similar level before and after stimulation (Fig. 4C and 4D), while Sp1 factor expression increased significantly after stimulation (Fig. 4E) thus confirming the results obtained by flow cytometry. It is worth noting that this is the first time that the presence of endogenously expressed GATA1 factor is described in CD4^+^ lymphocytes.

### Expression of *EPOR* Gene in Stimulated CD4^+^ Lymphocytes by Nested and Real-time PCR

**Figure 5:**
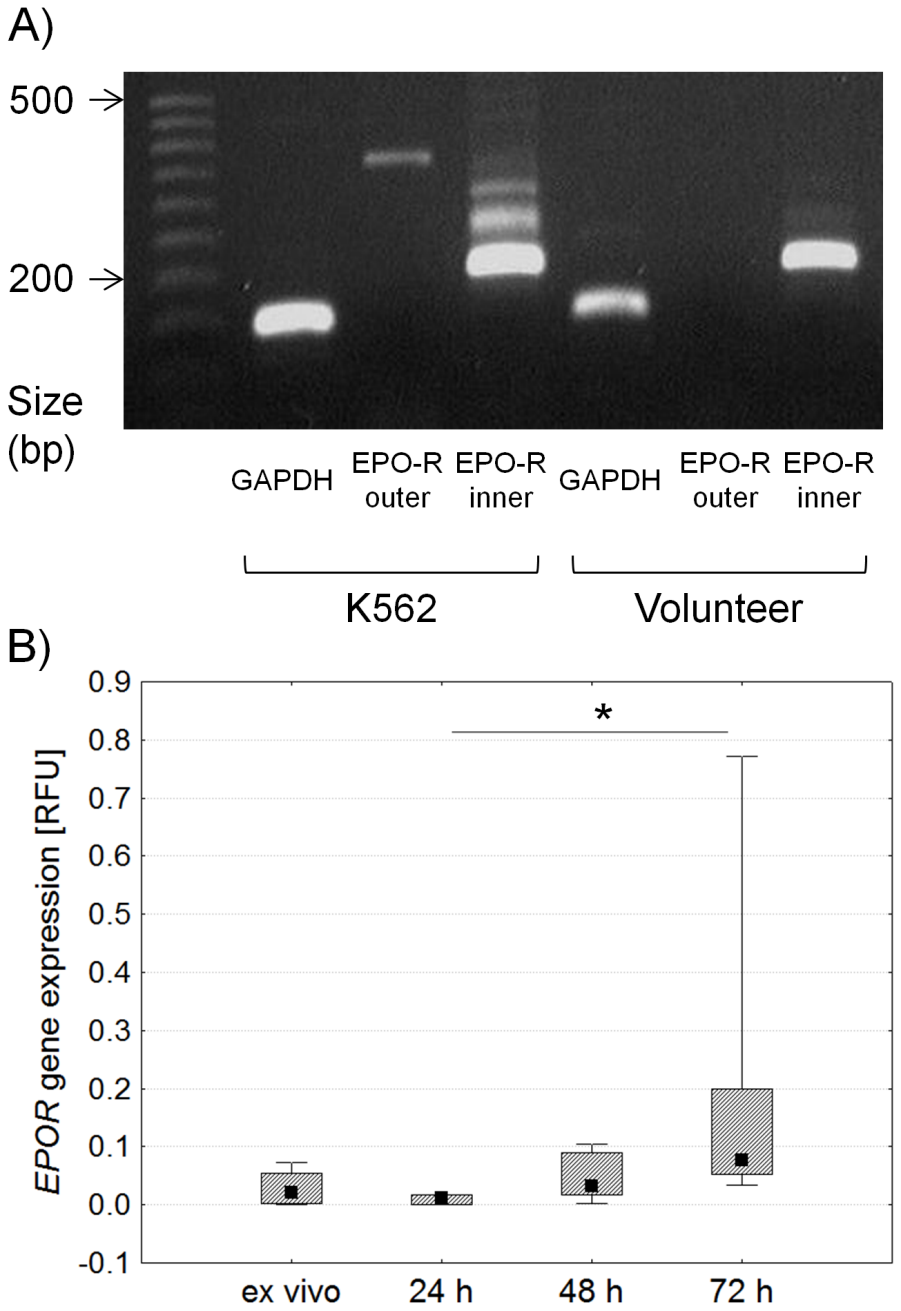
Estimation of *EPOR* gene expression by nested and real-time PCR in isolated CD4^+^ lymphocytes before and after stimulation with anti-CD3 antibody. Figure A presents *ex vivo* expression of *GAPDH* reference gene after a single PCR run and expression of *EPOR* gene after two runs of PCR in K562 cell line (positive control) and a healthy volunteer; the remaining five PCR results were similar to the ones shown. Figure B shows a comparison of *EPOR* gene expression determined by real-time PCR in isolated CD4^+^ lymphocytes before and after stimulation with anti-CD3 antibody. Midpoints in the figures present medians, boxes present the 25 and 75 percentile and whiskers outside visualize the minimum and maximum of all the data, *p<0.05, Friedman ANOVA and Post Hoc test, RFU – relative fluorescence units.

It should be stressed that in CD4^+^ cells the amount of the *EPOR* gene product is small compared to reference genes such as β-actin or GAPDH (3). Expression of EPOR gene in 600 000 magnetically separated CD4^+^ cells was undetectable after one run of PCR. Therefore, we employed nested PCR to detect the *EPOR* gene product in CD4^+^ lymphocytes before and after stimulation with anti-CD3 antibody (Fig. 5). Figure 5A shows the expression of *GAPDH* reference gene after a single PCR run in the context of *EPOR* gene expression after the first (EPO-R outer) and second (EPO-R inner) PCR run in a healthy volunteer. Since the amount of *EPOR* gene was too small to be estimated based on PCR alone we used the real-time PCR quantification method to measure the gene’s expression in CD4^+^ lymphocytes before and following 24, 48 and 72 hours of stimulation with anti-CD3 antibody. Expression of *EPOR* gene increased 72 hours after stimulation compared to the expression of *EPOR* gene 24 hours after stimulation (Fig. 5B).

**Figure 6:**
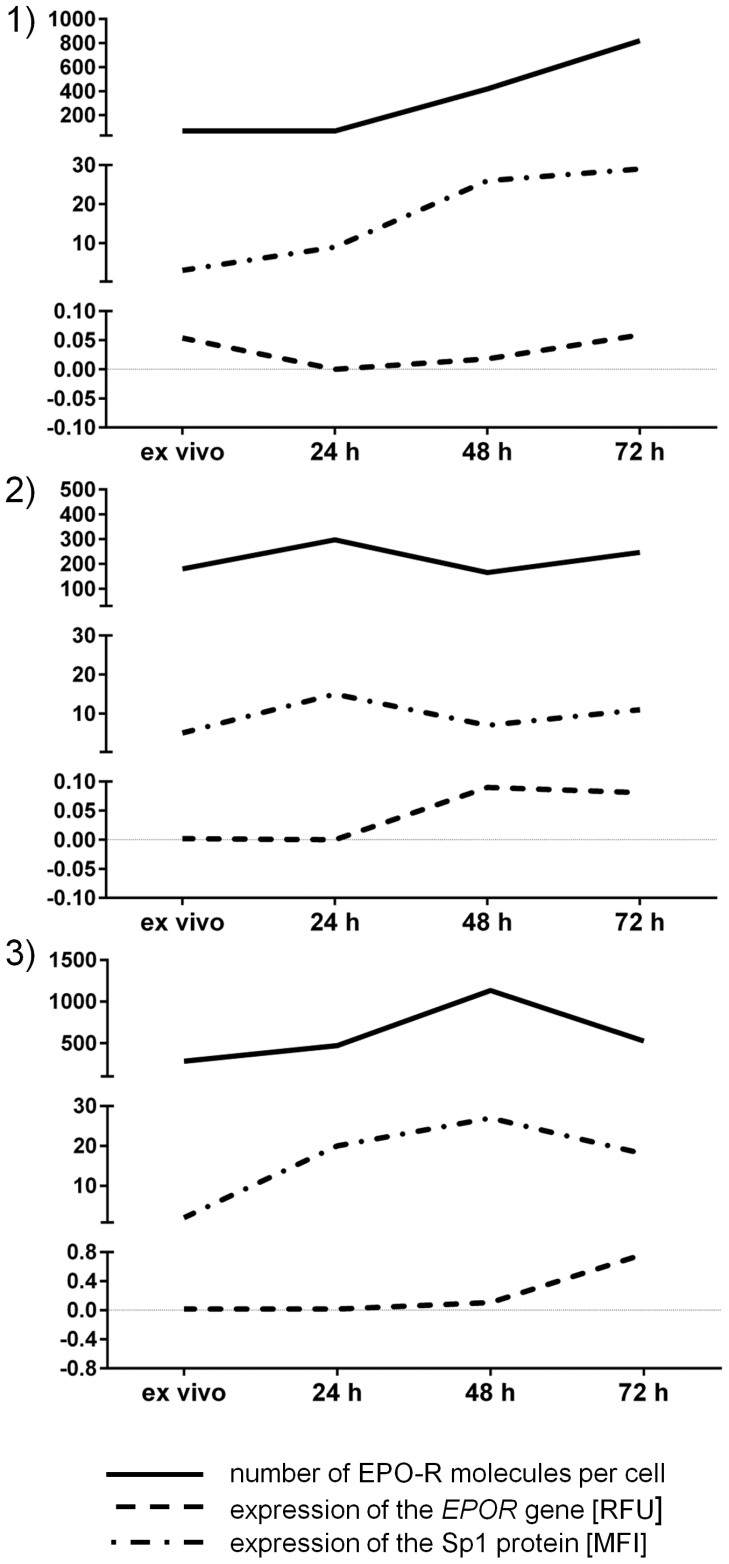
Changes in the number of EPO-R molecules per cell, *EPOR* gene expression and Sp1 expression in CD4^+^ lymphocytes before and after stimulation with anti-CD3 antibody. The figure shows results from 3 different individuals, MFI – mean fluorescence intensity, RFU – relative fluorescence units.

Figure 6 presents time-dependent changes in CD4^+^ lymphocytes after stimulation with anti-CD3 antibody regarding the following features: the number of EPO-R molecules per cell, the expression of the *EPOR* gene and the expression of Sp1.

## Discussion

Expression of EPO-R on the surface of granulocytes was first described a few years ago [20] pointing towards a possible role of EPO and its receptor in immune response. Moreover, we have recently presented evidence for a direct influence of rhEPO on CD4^+^ lymphocytes which modulates their signaling pathways by increasing phosphorylation of the signal transducer and activator of transcription 5 (STAT5) and modifying CD95 expression [4]. Although the level of EPO-R in CD4^+^ lymphocytes is very low (100 molecules per cell), stimulation with anti-CD3 antibody results in its increase to a value as high as 1000 molecules per cell [3] which is reported to be typical for erythroid cells [5]. The available literature indicates that the regulation of *EPOR* gene expression in erythroid cells depends on GATA and Sp1 transcription factors [11]. Therefore, we examined the expression of these transcription factors compared to the expression of EPO-R in human CD4^+^ lymphocytes stimulated with immobilized monoclonal anti-CD3 antibody.

We have shown that the number of EPO-R molecules on the surface of CD4^+^ lymphocytes significantly increases after stimulation with anti-CD3 antibody accompanied by the significant increase in the expression of Sp1 factor. We observed a strong positive correlation between the expression of Sp1 factor and the number of EPO-R molecules in stimulated CD4^+^ lymphocytes. An increased expression of Sp1 in stimulated CD4^+^ lymphocytes was supported by both Western Blot and flow cytometry analysis. We also detected GATA1 and GATA3 in isolated CD4^+^ lymphocytes using the Western Blot method, however their expression was at a similar level before and after stimulation. Admittedly GATA3 expression does appear to change as a result of stimulation, but these changes are not statistically significant. Expression of the GATA3 transcription factor has already been described in CD4^+^ cells mainly in the context of the cells’ differentiation toward the Th2 immune response. GATA-3 is expressed in peripheral naive CD4^+^ lymphocytes as well as in cells of the Th2 lineage [21]. GATA3 expression remains stable during the entire process of differentiation into Th2 cells but dramatically decreases after 2 days of priming for the Th1 response [21]. One must take into account that in cell culture T lymphocytes produce both Th1 and Th2 cytokines after stimulation with anti-CD3 antibody, which would explain these insignificant changes seen in GATA3 expression during stimulation.

Endogenous GATA1 expression has not yet been described in T lymphocytes. Therefore, its function and the regulatory mechanism of its expression in lymphocytes are unknown. A few years ago, Sundrud and colleagues showed that neither GATA1 nor GATA3 is expressed in purified, activated CD4^+^ cells unless they are transduced with HIV-derived vectors (HDVs) containing *GATA1* or *GATA3* genes [22]. These results are inconsistent with the results of our team and those of Wei-ping et al [21], which can be explained by different conditions of the experiments performed. We have detected endogenous expression of both GATA1 and GATA3 in isolated CD4^+^ cells of all healthy volunteers before and after stimulation. However, no other modifications of the cells’ environment was made than the use of immobilized monoclonal anti-CD3 antibody in order to stimulate the PBMCs; we did not use HDVs, which might interfere with normal cell functions.

The GATA transcription factors, whose levels does not change in stimulated CD4^+^ lymphocytes, could be responsible for maintaining a basic level of EPO-R in these cells. The single transcription factor, whose level changes as a result of CD4^+^ lymphocyte stimulation, is Sp1 factor. There seems to be a relationship between the expression of Sp1, the expression of the *EPOR* gene and the number of EPO-R on the surface of stimulated CD4^+^ lymphocytes. However, it has been shown in various cells that the activity of the *EPO-R* gene promoter is dependent on the integrity of both the GATA- and Spl-binding sites [23], [24]. In neuronal cells overexpression of GATA factors has no significant effect on *EPOR* mRNA expression since expression of *EPOR* in these cells gene is mainly dependent on the concentration of oxygen [16]. In cardiomyocytes Sp1 is essential for GATA-mediated *EPOR* gene transcription [24]. Furthermore, different GATA factors may be involved in regulating the expression of the *EPOR* gene, because these factors recognize the same DNA consensus sequence [17]. For example, co-expression of *GATA2*, *GATA3* and *EPO-R* genes is observed in astrocytes, while in microglia cells the *EPOR* gene is co-expressed only with GATA3 [16].

Our discovery that EPO-R is expressed on the surface of lymphocytes suggests that EPO might be capable of modulating or amplifying particular signaling pathways which are important for the cells’ functions. Previously, we have presented evidence for the direct influence of rhEPO on lymphocytes that can modulate their function. Our current study suggests that the expression of EPO-R in activated CD4^+^ lymphocytes is differentially regulated by GATA and Sp1 transcription factors. However, additional studies need to be carried out in order to determine which of these transcription factors is essential for regulating *EPOR* gene transcription in lymphocytes.
